# RDFizing the biosynthetic pathway of *E.coli* O-antigen to enable semantic sharing of microbiology data

**DOI:** 10.1186/s12866-021-02384-y

**Published:** 2021-11-22

**Authors:** Sunmyoung Lee, Tamiko Ono, Kiyoko Aoki-Kinoshita

**Affiliations:** Graduate School of Engineering; Glycan and Life Systems Integration Center, Soka University, Hachioji, Tokyo 192-8577 Japan

**Keywords:** *E.coli* O-antigen, RDF, BioPAX ontology, SPARQL, RDFization, Semantic data

## Abstract

**Background:**

The abundance of glycomics data that have accumulated has led to the development of many useful databases to aid in the understanding of the function of the glycans and their impact on cellular activity. At the same time, the endeavor for data sharing between glycomics databases with other biological databases have contributed to the creation of new knowledgebases. However, different data types in data description have impeded the data sharing for knowledge integration. To solve this matter, Semantic Web techniques including Resource Description Framework (RDF) and ontology development have been adopted by various groups to standardize the format for data exchange. These semantic data have contributed to the expansion of knowledgebases and hold promises of providing data that can be intelligently processed. On the other hand, bench biologists who are experts in experimental finding are end users and data producers. Therefore, it is indispensable to reduce the technical barrier required for bench biologists to manipulate their experimental data to be compatible with standard formats for data sharing.

**Results:**

There are many essential concepts and practical techniques for data integration but there is no method to enable researchers to easily apply Semantic Web techniques to their experimental data. We implemented our procedure on unformatted information of *E.coli* O-antigen structures collected from the web and show how this information can be expressed as formatted data applicable to Semantic Web standards. In particular, we described the *E-coli* O-antigen biosynthesis pathway using the BioPAX ontology developed to support data exchange between pathway databases.

**Conclusions:**

The method we implemented to semantically describe O-antigen biosynthesis should be helpful for biologists to understand how glycan information, including relevant pathway reaction data, can be easily shared. We hope this method can contribute to lower the technical barrier that is required when experimental findings are formulated into formal representations and can lead bench scientists to readily participate in the construction of new knowledgebases that are integrated with existing ones. Such integration over the Semantic Web will enable future work in artificial intelligence and machine learning to enable computers to infer new relationships and hypotheses in the life sciences.

**Supplementary Information:**

The online version contains supplementary material available at 10.1186/s12866-021-02384-y.

## Background

Glycomics data that have been accumulated based on high-throughput techniques have revealed the importance of the biological roles of glycans across cellular activities, such as signal transduction [1], cellular proliferation [2], and immune recognition [3] as well as energy generation [4]. These studies have led to the development of many valuable databases in glycobiology. The Consortium for Functional Glycomics (CFG) has provided information to help in the elucidation of the relationships between glycan structure and function at the level of glycan-protein interactions in cell-to-cell communication [5]. In KEGG GLYCAN, glycans are presented in biological pathway maps, and each glycan structure and relevant enzyme information can be identified [6]. GlycoGene DataBase (GGDB) has provided information of human and mouse glycogenes involved in glycan synthesis such as glycosyltransferases, transporters, and sugar nucleotide synthetases [7]. GlyTouCan has been developed as the international repository for glycan structures, assigning unique accession numbers to each identified glycan [8], which allows complex carbohydrate structures to be referenced within the biological community without confusion or ambiguity, and as such serves as an essential element for sharing of glycan data between different databases. With these advances in glycan database development, efforts for data sharing between different major databases have been conducted [9]. The resources containing biological information are shared between not only glycan-related databases but also with other biological databases such as biological pathway databases, thus contributing to the creation of new knowledgebases. Such knowledgebases developed by integration or sharing of data instead of the accumulation of individual data types can help researchers investigate glycan function in the context of complex biological processes; they can also contribute to reduce the time and effort consumed on searching for data scattered across many kinds of databases. For data sharing, the bottom line is standardization of incompatible data formats, which enable data to be interpreted by computational means. Through this process, resources that contain fragmented information can be made to easily collect, interpret, and share data across different databases.

Semantic Web Technologies have been adopted by major databases in the life science fields [10, 11]. This technique has evolved to integrate heterogeneous representations of data mainly through the development of standardized ontologies, which can formally express knowledge in a particular domain [12], and Resource Description Framework (RDF). RDF is a framework for recording data in a machine-readable format and describes a data model. It represents information about resources (data items) using triple statements, which are composed of subject, predicate (representing a “property” of the subject), and object (representing the “value” of the property). All three components of these triples are represented by either universal identifiers (i.e., Internationalized Resource Identifiers (IRIs)) or as literal values (for objects). Therefore, all resources represented in RDF format can be explicitly identified without ambiguity. The data represented in such a structured and standardized format can be easily linked with other RDF data.

More importantly, these data resources are annotated with information about their semantics, or meaning, via the use of predefined ontologies. For example, the Gene Ontology [13] has been used to annotate information about gene function, molecular properties and cellular localization, allowing for easier enrichment analysis of gene microarray experiments [14]. Thus, with this data description format of RDF data combined with ontologies, resources that are described with high variability can be represented using a common or collective vocabulary. In particular, in the current microbiology landscape, the formats and annotations of biological data are too heterogeneous to share and integrate because of their intrinsic complexity. Therefore, ontologies defined within a specified knowledge domain can be a solution to resolve such ambiguity originating from the variety of knowledge representations.

Ontologies consist of a description of (a) things or concepts and (b) relationships between two things or concepts, which are called **Classes** and *properties*, respectively [15]. **Classes** represent specific concepts, such as genes and compounds, whereas *properties* are associated with specific **Classes** and describe the relationship between two **Classes** or between a **Class** and a literal value. For example, a gene **Class** may have a gene ID associated with it, in which case the *property* may be something like “has_geneID” and its object would be a literal value. Alternatively, a gene **Class** could be associated with the protein it encodes, in which case a *property* such as “encodes_protein” may be defined to associate a gene **Class** with a protein **Class**. Once these ontological terms are defined, then RDF data can be generated to associate meaning with actual data. Often, an ontology may already be defined by others, in which case existing ontologies should be used wherever possible in order to avoid duplication of effort. Databases of such ontologies, such as BioPortal [16] and the OBO Foundry [17], can be searched to find the most appropriate ontology.

Many ontologies have been developed in the biomedicine field, and in this work, since we wanted to describe a biosynthetic pathway, we decided to use the BioPAX ontology which is a strong community-based and standardized ontology developed for biological concepts; BioPAX has been designed in particular for pathway descriptions and data exchange between biological pathway databases [18]. The BioPAX ontology has been considered as one of the most successful of standardized languages as it facilitates the exchange of biological pathway data at the molecular and cellular levels, and as such it has been adopted by major databases such as Reactome [19], BioCyc [20], WikiPathways [21], etc.

In this study, O-antigens found in the outermost membrane of *E. coli* were targeted for semantic representation. Many studies have documented that O-antigens composed of polysaccharides are responsible for causing a broad spectrum of diseases in humans and animals, and it is known that the O-antigen serogroup provides information about the pathogenicity of *E.coli* [22]. However, there is no available semantic data allowing reuse of information associated with this glycan antigen. We attempted to present the glycan structures of *E. coli* O-antigens semantically, represented in a standard format with the BioPAX ontology. As a result, we show that they can be successfully represented in a structured data format using the BioPAX ontology and basic tools and techniques that any biologist can master. This study introduces a straightforward method for RDFization of unstructured glycan information on the web. Our methods for RDFization will help researchers who are not familiar with Semantic Web technology to handle their experimental findings and transform them into interoperable semantic data.

## Results

### RDF modeling for the biosynthesis pathway of *E. coli* O-antigen structures

**Fig. 1:**
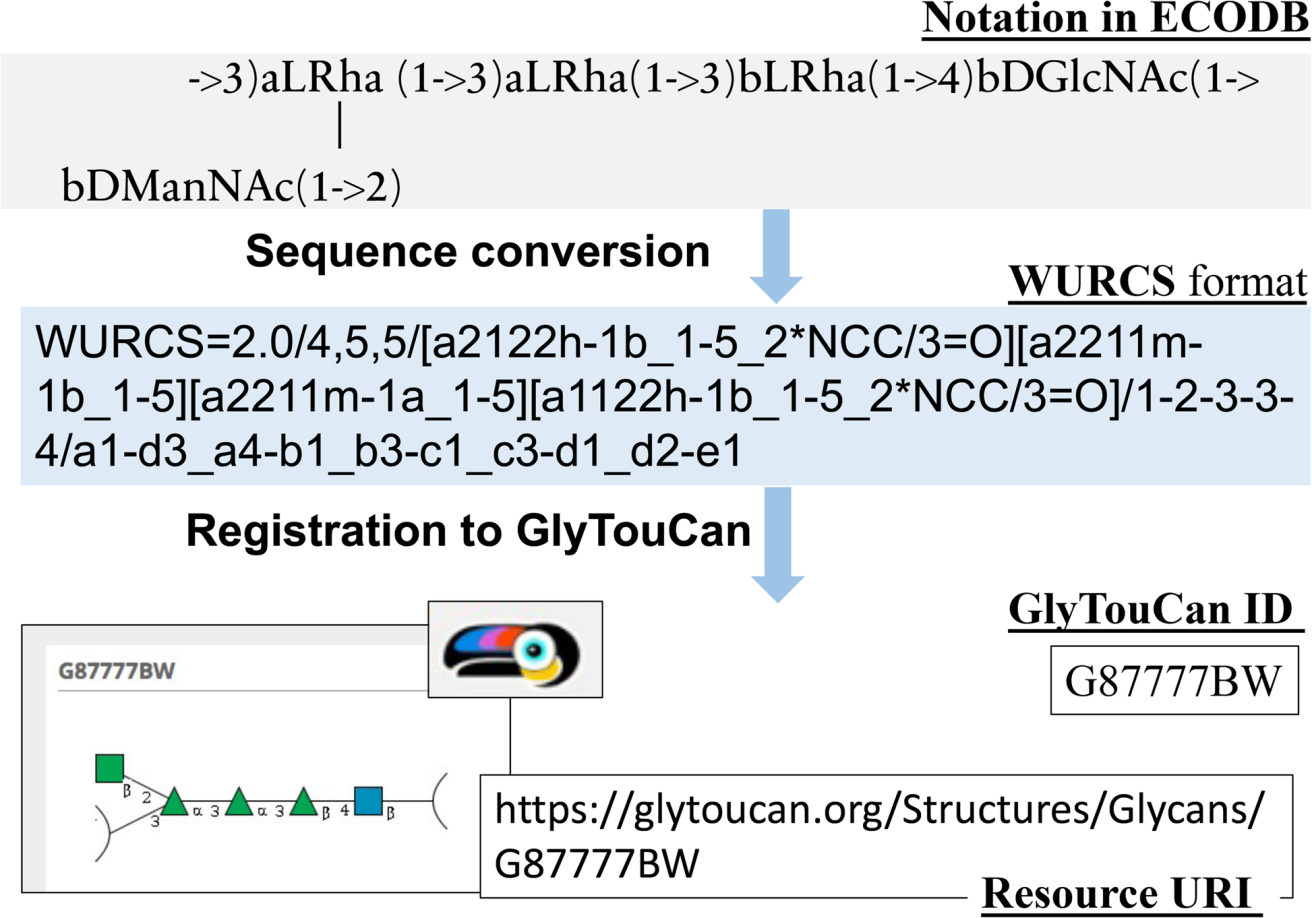
Example of acquiring the GlyTouCan ID of an O-antigen structure (*E.coli***O1A**). To assign unique accession numbers to all glycan structures, a list of O-antigen collected from ECODB were converted WURCS format and then registered to GlyTouCan, the international repository for glycans, which allowed every glycan to be referenced by a unique ID without confusion

In this work, we modeled the biosynthetic pathway of *E. coli* O-antigen structures. The necessary information to describe this were collected from external databases on the Web, and we designed an RDF model using an ontology while taking into consideration the provenance and connectivity to other online data resources. Metadata information about the *E. coli* O-polysaccharide antigen such as the list of glycans, corresponding glycosyltransferases, and literature references were collected from ECODAB (*E.coli* O-antigen database) [23] and CSDB (Carbohydrate structure database) [24]. To standardize the notation of complex carbohydrate structures, donor monosaccharides, intermediate saccharides occurring during the biosynthetic process of O-antigen, and complete O-antigens, these structures were converted to WURCS (Web 3.0 Unique Representation of Carbohydrate Structures) [25] format. These glycans were registered in the glycan repository GlyTouCan in order to obtain a unique accession number to use across all resources, which can be used as an IRI to maintain consistent referencing of glycan structures (Fig. 1) Therefore, all saccharides in this study were assigned a unique GlyTouCan accession number and IRI which is composed of the prefix, “http://glytoucan.org/Structures/Glycans/”. By using this IRI pattern for glycans, it became straightforward to link glycans with other data without concern for whether glycan resources can be accessed or where they should be referenced. In other words, these IRIs could be used as the standard identifier for glycans at a global level.

**Fig. 2:**
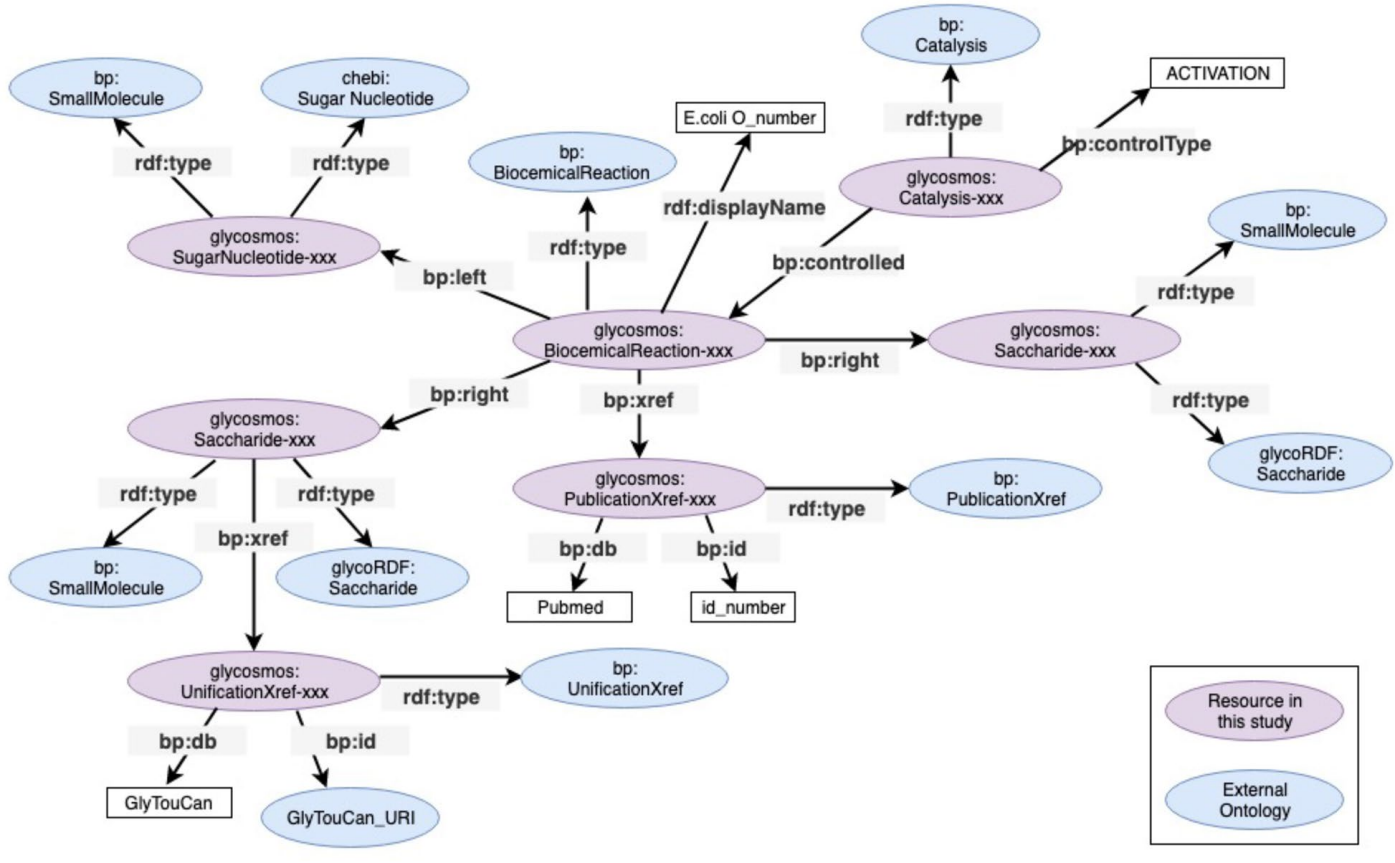
RDF graph model for representation of O-antigen synthetic pathway. A biochemical reaction as part of our O-antigen RDF graph model consists of glycan linkages formed by a glycosyltransferase. ‘bp:’ and ‘rdf:’ are abbreviations of the namespaces for BioPax (http://www.biopax.org/release/biopax-level3.owl#) and RDFS-Schema (http://www.w3.org/1999/02/22-rdf-syntax-ns#), respectively

The RDF graph model of O-antigens was described using RDF Schema (RDFS) [26] and terms from the BioPAX ontology, which provide the necessary vocabulary terms for describing basic cellular process such as interactions, reactions, and information order between molecules in biological pathways. Using this ontology, the O-antigen structure could be represented as a pathway proceeded by a series of catalytic reactions. The pathways for O-antigen synthesis consist of reactions that represent a glycosidic linkage between a glycan substrate and product glycan. Each reaction can be described using (a) glycans describing donors (sugar nucleotides), acceptor (substrate glycan), and product glycan and (b) glycosyltransferases. To implement this concept with the BioPAX ontology, we designed an RDF model. Figure 2 illustrates this model, which is shown as a data schema and is a crucial step in expressing the intended pathway correctly. In this figure, the property *rdf:type* defines the class of the object being referenced; an object may be defined using multiple *rdf:type*s depending on the context by which the data is accessed. Based on BioPAX rules (indicated by the prefix “bp:”), each reaction forming a glycosidic linkage was represented using the **bp:BiochemicalReaction** class. This class describes the reactant, consisting of a donor and acceptor glycan, and a glycan product using the *bp:left* and *bp:right* properties, respectively. To define instances of this class as reactions forming a glycosidic bond, the sugar nucleotide of the reactant was assigned the **SugarNucleotide** class of the ChEBI (Chemical Entities of Biological Interest) ontology [27], and extended glycans and the resulting product glycan were represented as both a **Saccharide** class of the GlycoRDF ontology [28] and a **SmallMolecule** class of the BioPAX ontology. The **BiochemicalReaction** class was described as a reaction controlled by an entity of the **Catalysis** class using the *bp:controlled* property. The **Catalysis** class was used to express the activity of glycosyltransferases using the bp:*controller* property.

To specify the order of multiple reactions, all reactions were sequentially ordered using the **BiochemicalPathwayStep** class and the *nextStep* property. This class is linked to the **Pathway** class by bp:*pathwayOrder* properties, which can specify consecutive (glycan) reactions. Additional information about each instance such as specific glycans or enzymes are described using the bp:*xref* property; for example, the relevant literature and GlyTouCan accession numbers were provided using the **PublicationXref** and **UnificationXref** classes, respectively. In the case of **UnificationXref**, this is a class for aiding data integration and its usage is highly recommended. Using this class would help reduce efforts in data sharing between diverse databases. Detailed descriptions about these properties and classes used in this study is listed in Additional file 1, where each class and its associated predicates are described along with examples of their usages in Turtle format [29], a common representation format for RDF data.

### Data arrangement into table format

**Fig. 3:**
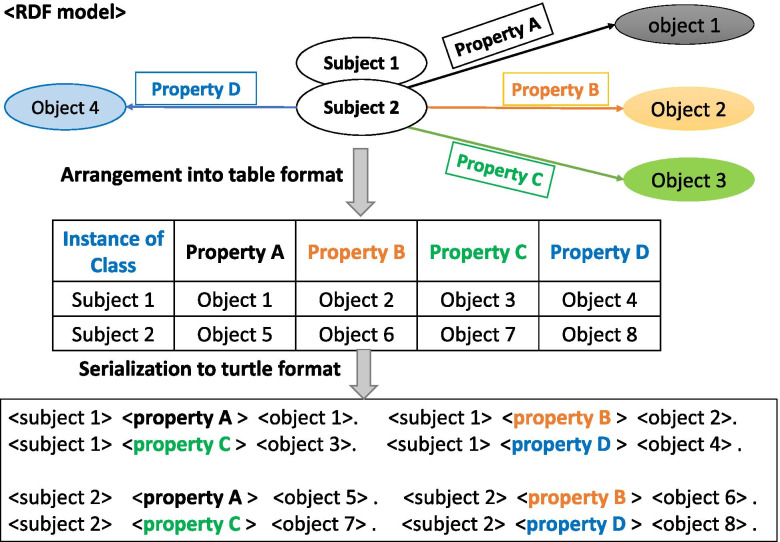
Arrangement of data for RDFization. Once a data model and relevant ontologies have been established, the data at hand should be arranged in table format to be transformed into RDF triples. Subjects and objects represent resources, which are connected by properties. Detailed information about resources, or specific data types, can be expanded by including the proper properties and creating triples to link the details with the data

Because of the triple format of RDF, RDF data can be described as graphs, where nodes are connected by edges. A node of an RDF graph corresponding to a subject or object becomes an instance of a class in the corresponding ontology. To apply this RDF model to the *E.coli* O-antigen pathway, all subject instances that belonged to the same class were arranged in the leftmost column of a spreadsheet table. For properties that define a relationship between a subject and object, property statements were listed in the first heading row of the table to use in generating the triple graph for RDFization. The property values corresponding to instances of object classes are placed in a cell of the relevant column corresponding to its property. This rearrangement of data into a table form makes it easy for a researcher to manage data and avoids the necessity to use another specialized software for creation of instances. Figure 3 illustrates this method showing how RDF data should be arranged in a table form for triple generation. In this figure, subjects and objects are instances of **Class** that can be grouped together into meaningfully same resources. For example, O-antigen subunits of *E.coli* serogroups such as O-1, O-2 can be grouped as **Class** named **Saccharide** and each individual O-antigen become instance of this class.

### RDF serialization of O-antigen information in a table

**Fig. 4:**
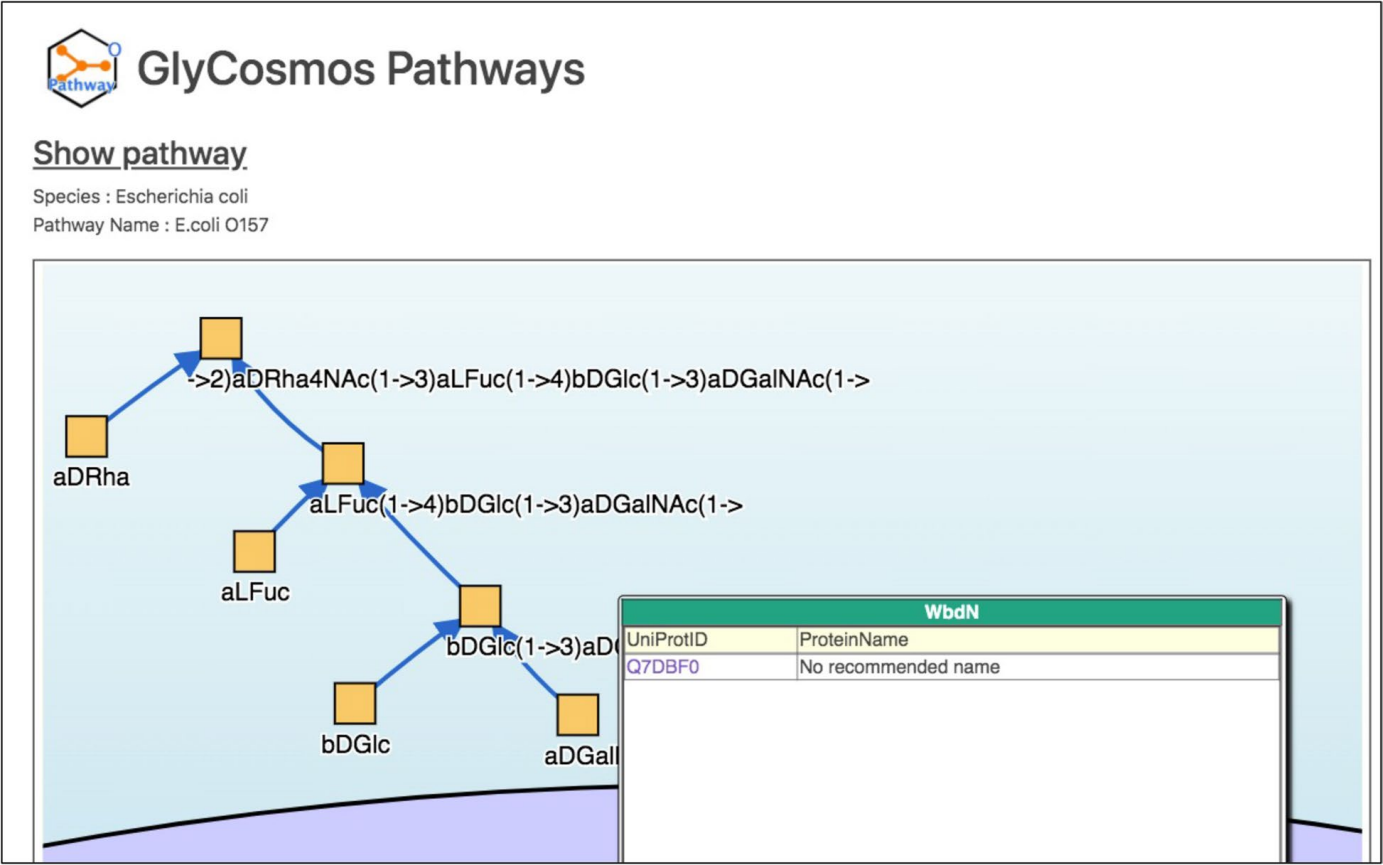
Visualization of the O-antigen pathway in GlyCosmos Portal sites (http://glycosmos.org). The data transformed to RDF format can be easily visualized in a Semantic Web-supporting database, such as Virtuoso [33]. User can identify the enzyme information responsible for glycosidic bond when cursor is hovered over blue line with an arrowhead

To convert data arranged in a table format into RDF statements, we used *RDFLib* which is a python package assisting RDF data handling [30]. The table data stored as a csv file were transformed into triple sentences by our python code. The source code for transforming csv files to triples is available as open source code at https://gitlab.com/lsunmyoung/rdfization (with the version 2 used for the data described in this manuscript available on zenodo at https://zenodo.org/record/5587751#.YXDd59lBz0o). A more specific procedure for RDF documentation is explained in the Methods section and Fig. 7. Using this code, RDF triples for describing *E.coli* O-antigen structures from O1 to O188 could be generated in Turtle format which is the most human-readable text format for RDF among other syntax formats. The triples were loaded into a Virtuoso database server, and the validity of the data was confirmed in our SPARQL endpoint (https://ts.glycosmos.org/sparql), where the data can be queried using the SPARQL query language (described in the next section) (Additional file 3) [31]. Finally, the user interface to display this uploaded RDF data was developed using a visualization tool. This data, among others, can be viewed from the GlyCosmos Portal (https://glycosmos.org) by searching for *E. coli* O-157 (Fig. 4) in the GlyCosmos Pathways dataset [32].

### SPARQL queries on the RDFized *E.coli* O-antigen biosynthesis pathway data

Using the validation service provided by the W3C, we confirmed that there were no syntactic errors in our RDF syntax, and the visualized graph correctly showed the relationships between classes via their properties. SPARQL (SPARQL Protocol and RDF Query Language) is a query language for data in RDF stores which are arranged as graphs [34]. SPARQL queries for questions that we want to answer are also composed of triples, which provides us a way to inspect specific information stored in the RDF data in detail [35, 36]. In this study, SPARQL queries were designed to evaluate the RDF data generated by our method. Table 1 lists a few examples of SPARQL queries on our RDF data and illustrates how a SPARQL query can be made to retrieve relevant data. The “PREFIX” is declared using abbreviations of the full namespace IRI pointing to an RDF document where the vocabularies are already defined. All resources within the same RDF document are written with a prefix followed by a colon (:). An example of a prefix for the BioPAX ontology IRI is as follows:

**Table 1:**
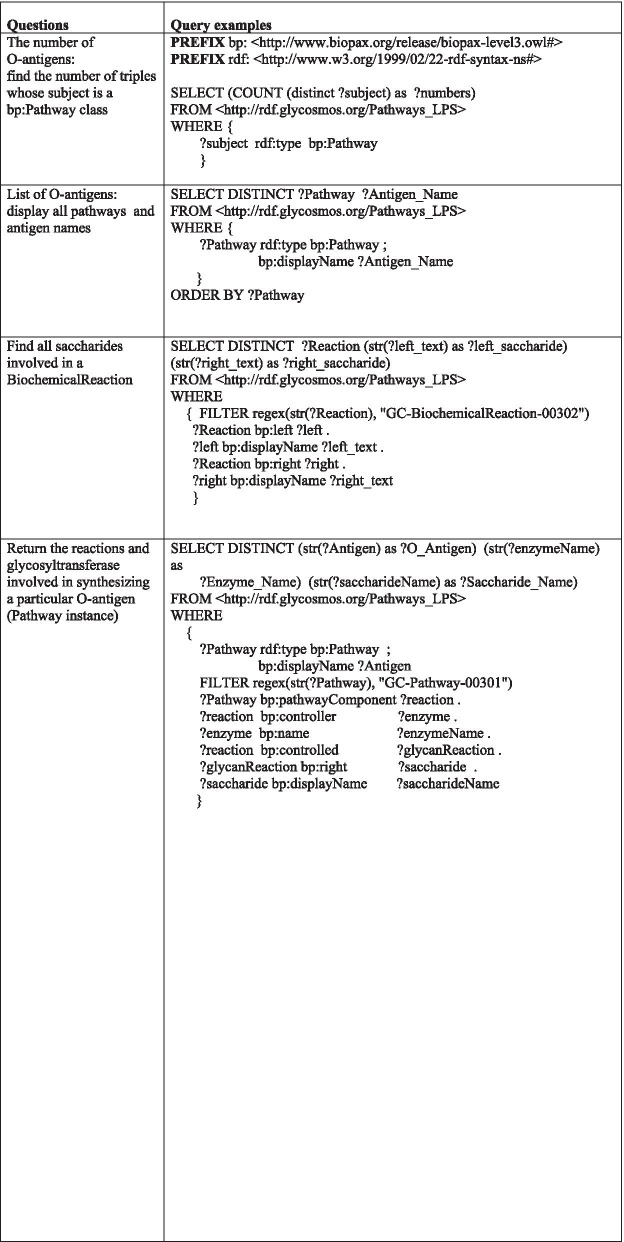
Example queries for confirmation of the uploaded RDF triples (PREFIXs are common to all Query examples)

**PREFIX** bp: <http://www.biopax.org/release/biopax-level3.owl#>

**Fig. 5:**
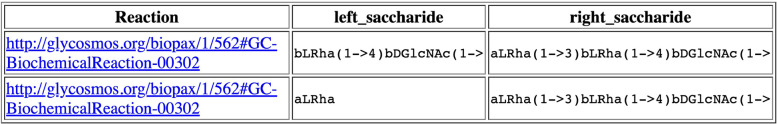
Query result of 3rd question in Table 1. Result against 3rd query of Table 1 shows reactant polysaccharide, donor sugar nucleotide, and product polysaccharide, which are component molecules of a reaction instance (“GC-BiochemicalReaction-00302”)

**Fig. 6:**
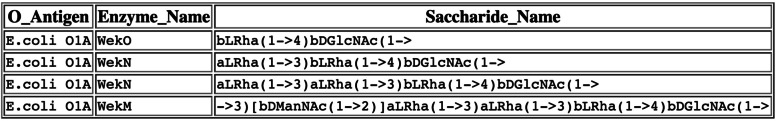
Query result of 4th question in Table 1. Result against 4th query of Table 1 shows glycosyltransferase and polysaccharides that compose an arbitrary O-antigen, O1A in this query

The values that we want to retrieve are stored in variable names which start with “?”. The first and second queries were designed to identify the number and name of *E.coli* O-antigens stored in our triplestore. Note that the predicate “a” is used as an abbreviation to refer to the “rdf:type” predicate. The retrieved results showed 192 O-antigens together with the names of each O-antigen (Additional file 2). In order to validate the composition of our **BiochemicalReaction** instances described by *bp:left* and *bp:right* properties, the third SPARQL query was created. Our question was whether one saccharide corresponding to a product in a glycosidic reaction is composed of a sugar nucleotide and reactant saccharide that is deficient by one monosaccharide. An arbitrary instance of a specific reaction (GC-BiochemicalReaction-00302) was selected by adding a keyword “FILTER” to the query (Table 1). This query returned the correct answer as we predicted (Fig.5). The fourth query was designed to identify the glycans and glycosyltransferases that make up a pathway under the constraints that the name is “O-antigen 1A” and the name of the **Pathway** is “GC-Pathway-00301”. To limit to O-antigen O1A, a regular expression (regex) filter term was used as in the third query and then the name of the “**Pathway**” was set to “GC-Pathway-00301”. As confirmed in Fig. 6, the O1A antigen is composed of four intermediate glycans synthesized by four glycosyltransferase enzymes, validating our data by showing that the correct results could be retrieved as well. In summary, we were able to show that RDF triples about *E.coli* O-antigens could be successfully constructed as a pathway process using BioPAX ontologies.

## Discussion

The BioPAX ontology was created to facilitate exchange of many fragmented pathway data in heterogeneous formats from multiple databases. Because this ontology is supported by a strong community, major public pathway databases such as WikiPathways [37], Reactome [38], BioModels [39] etc. have been developed using this ontology as a standard. As glycans have key roles in important pathway events in multicellular organisms [40], integration of glycan information with pathway data resources will lead to a more comprehensive understanding of cellular function. Considering the exploding amount of data regarding both glycans and pathways among the literature, because such data is available only as text, standardization of data into a computer-readable format can contribute to accelerated data sharing. In this work, we showed the method of data standardizations that the bench biologist can easily implement. Here, we represented *E. coli* O-antigens using standard ontologies and RDF format. In addition to BioPAX, we considered ontologies in the glycobiology field such as GlycoRDF [28], Glycomics Ontology [33], and GlycoConjugate Ontology [41] which are already developed and used. However, these ontologies are aimed at representing only glycan or glycoconjugate structures, and none of these were suitable to capture the glycan structure to be represented in a glycosylation pathway. We hope that through this manuscript, we provided a straightforward method to reconstruct biological information (in our case *E.coli* O-antigens) existing in unstructured and heterogeneous formats into a more standardized format using Semantic Web technologies. After RDFization, we could validate our generated RDF triples of O-antigens through results retrieved by SPARQL queries. The SPARQL query was executed in Virtuoso, a graph database for RDF triples, which is appropriate for data integration as they enable multiple data sources to be combined into a single graph. If our RDF data is cross-referenced with UniProt [42], which contains disease information, a more detailed question, for example, “Which O-antigens are related to diarrhea?” can be answered by executing a federated SPARQL query over the SPARQL endpoint of UniProt together with that in GlyCosmos. In other words, such queries can be made simultaneously across multiple SPARQL endpoints without the need for transferring large amounts of data to compare. This implies that O-antigen information constructed in RDF format can be easily expanded through sharing or integration with other structured data resources in RDF format and therefore we can get answers to more complex biological questions. Alongside this SPARQL query, the visualization of the ontology can help researchers unfamiliar with ontologies to better understand more complex ontologies using tools such as Visual Notation for OWL Ontologies (VOWL) [43], which provides diagram representations of classes, properties, and datatypes (http://www.visualdataweb.de/webvowl/).

As one method for standardization of data, we have implemented a simple process for RDFization of unstructured data and described key concepts and terms required to apply Semantic Web technologies. When we consider the data explosion and heterogeneous characteristics of data produced by high throughput technologies in the omics fields, representation of data by standardization is essential for construction of a new knowledgebase through data exchange with the other databases implemented in not just a computer-readable, but also a computer-understandable format. Our method will be a beneficial guide to reducing the consuming time and effort needed to get acquainted with complex programming tools. In addition, this will lower the barrier for biological researchers to directly take part in the construction of new knowledge integration reflecting their experimental data.

## Conclusions

To facilitate data sharing we presented an effective method for the standardization of unstructured data. Our method is designed to show that data that is available only in the scientific literature and which a computer is not able to understand can be integrated with pathway information; this method should help lower the technical barrier required for experimental biologists to create and maintain semantic data. We showed how the unformatted data should be organized in a table format and the usage of controlled vocabularies such as the BioPAX ontology which are well-defined to represent pathways in a computable format. The application of our method to *E. coli* O-antigens can be applied to describe other bacterial O-antigens, so that these expanded data can enable comparisons across microbes. Future work will be implemented on glycans on bacterial outer membrane playing an important role in interactions between host and pathogen. We anticipate that the organized data in a computable format can contribute to the acceleration of integration of knowledgebases providing comprehensive understanding and insight into cellular activities.

## Methods

### Collection of O-antigen resources

Beautiful Soup, a python library, was used to collate the O-antigen list from the *Escherichia coli* O-antigen Database (ECODAB) which is dedicated to *E.coli* O-antigen structures. This O-antigen list was translated to a linear text with an in-house code using the Python programming language (Python Software Foundation, http://www.python.org/), version 3.7.0 and then transformed to WURCS format, using the GlycanFormatConverter API provided by the GlyCosmos Portal, to register in GlyTouCan. The glycosyltransferase information needed to map with glycan linkage information was obtained from the Carbohydrate Structure Database (CSDB), which is a curated database developed for providing glycan information in prokaryotes, plants and fungi [24].

### Importing of existing ontologies

To represent the RDF model for O-antigens, the BioPAX ontology was used. An ontology generally consists of instances, classes, and properties: instances such as O1A, O4, O157 are objects within same class of named O-antigen; classes are used to represent concepts or objects; and properties are used to express relationships between two individuals or concepts. Documentation [18] provided by the BioPAX Working group was referenced for usage of each **Class** and *property* for describing concepts such as biochemical reactions, catalysis and pathways. To represent a glycan resource and information related with it, external ontology terms were imported such as **SugarNucleotide** and **Saccharide**, from ChEBI and GlycoRDF, respectively, because the BioPAX ontology does not support glycan-related vocabulary such as these.

### Serialization of O-antigen information

**Fig. 7:**
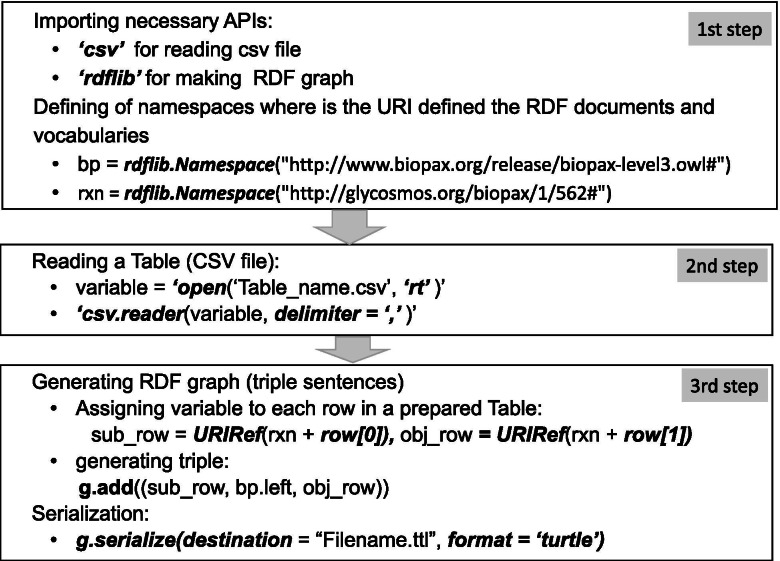
Code flow for RDFization in python language. This represents code flow for RDFization of table data in csv file format. The specific source codes are opened in this site (https://gitlab.com/lsunmyoung/rdfization). First step of coding for rdfization of table format data is to import necessary modules and define IRI of resources. Second step is to specify subject, property, and object of all instances in the tabular format. By the 3rd step, RDF sentences is produced with an intended the RDF format such as Turtle

The data arranged in table format according to our RDF data model for O-antigen needed to be transformed into RDF sentences. There are various formats for RDF documents such as N3, N-Triples, RDF/XML, and Turtle. Among these, RDF sentences in this study were represented in Turtle format because it is the most human-readable and concise. This allowed us to store the RDF data in a compact textual form, in which a long and repeated IRI can be written as a short prefixed name. To extract RDF triples from all instances in the same table file, we used the RDFLib python package [28]. The python code for serialization is mainly composed of three parts: importing a necessary API (Application Programming interface) from the ***importlib*** module, reading a csv file and creating nodes relevant to the **Classes**, adding instances to the RDF triple graph, and serialization. The more detailed code is explained in Fig. 7. The extracted RDF triples were loaded into a Virtuoso triple store [40], which is a graph database suited for storage of RDF data, and inspected via a SPARQL endpoint.

### Validation of RDF data

To inspect the structure and syntax of the RDF triples and to check whether our RDF/Turtle document was correct, we used a validation service developed by the W3C (http://www.w3.org/RDF/Validation), which can check for syntax errors in an RDF document and provide a visualization graph of the data. In addition to this validation, for inspecting the constructed RDF graph, SPARQL queries were made, and the results were evaluated to assess the accuracy of the stored RDF data (Table 1).

### Loading RDF triples into the Virtuoso database server

RDF triples have to be stored in an RDF store such as the Virtuoso database server in order to be processed and queried. To start up the service provided by virtuoso, the open-source version of the Virtuoso server program was downloaded and installed (virtuoso.openlinksw.com). After installation, the location at http://localhost:8890/ can be opened to access the Virtuoso Conductor page, from which data can be queried and uploaded. To upload, after logging in, navigate from “Linked Data” to Quad Store Upload” in the Web interface. RDF data file can be uploaded here. The uploaded RDF data can be accessed at https://ts.glycosmos.org/sparql. Through this SPARQL endpoint, integrated queries across all datasets in the Virtuoso server can be executed. Also all query results can be confirmed at https://ts.glycosmos.org/sparql, the SPARQL endpoint using the LODEStar linked data browser from the Functional Genomics Production Team (FGPT). Moreover, federated queries combining the local Virtuoso server with publicly available SPARQL endpoints such as UniProt are then possible.

## Supplementary Information

**Additional file 1: Table 1.** Usage of BioPAX vocabulary for *E*.*coli* O-antigen biosynthetic pathway description.

**Additional file 2. **List of *E*.*coli* O-antigens obtained from constructed RDF triples.

**Additional file 3. **SARQL queries to confirm the RDF data in endpoint and results.

## Data Availability

All source codes can be obtained from https://gitlab.com/lsunmyoung/rdfization. The version used in this work is available from zenodo (Version 2): https://zenodo.org/record/5587751#.YXDd59lBz0o.

## Abbreviations

WURCS: Web 3.0 Unique Representation of Carbohydrate Structures
RDF: Resource Description Framework
SPARQL: SPARQL Protocol and RDF Query Language
*E.coli*: *Escherichia.coli*
